# Two-Component Signal Transduction Pathways Regulating Growth and Cell Cycle Progression in a Bacterium: A System-Level Analysis

**DOI:** 10.1371/journal.pbio.0030334

**Published:** 2005-09-27

**Authors:** Jeffrey M Skerker, Melanie S Prasol, Barrett S Perchuk, Emanuele G Biondi, Michael T Laub

**Affiliations:** **1**Bauer Center for Genomics Research, Harvard University, Cambridge, Massachusetts, United States of America; Lawrence Berkeley National LaboratoryUnited States of America

## Abstract

Two-component signal transduction systems, comprised of histidine kinases and their response regulator substrates, are the predominant means by which bacteria sense and respond to extracellular signals. These systems allow cells to adapt to prevailing conditions by modifying cellular physiology, including initiating programs of gene expression, catalyzing reactions, or modifying protein–protein interactions. These signaling pathways have also been demonstrated to play a role in coordinating bacterial cell cycle progression and development. Here we report a system-level investigation of two-component pathways in the model organism *Caulobacter crescentus*. First, by a comprehensive deletion analysis we show that at least 39 of the 106 two-component genes are required for cell cycle progression, growth, or morphogenesis. These include nine genes essential for growth or viability of the organism. We then use a systematic biochemical approach, called phosphotransfer profiling, to map the connectivity of histidine kinases and response regulators. Combining these genetic and biochemical approaches, we identify a new, highly conserved essential signaling pathway from the histidine kinase CenK to the response regulator CenR, which plays a critical role in controlling cell envelope biogenesis and structure. Depletion of either *cenK* or *cenR* leads to an unusual, severe blebbing of cell envelope material, whereas constitutive activation of the pathway compromises cell envelope integrity, resulting in cell lysis and death. We propose that the CenK–CenR pathway may be a suitable target for new antibiotic development, given previous successes in targeting the bacterial cell wall. Finally, the ability of our in vitro phosphotransfer profiling method to identify signaling pathways that operate in vivo takes advantage of an observation that histidine kinases are endowed with a global kinetic preference for their cognate response regulators. We propose that this system-wide selectivity insulates two-component pathways from one another, preventing unwanted cross-talk.

## Abstract 2

Histidine kinases and their (sensory) response regulators are screened for in *C. crescentus.* Follow-up experiments determine several essential components, including one pair critical for cell envelope biogenesis and structure.

## Introduction

Cells have the remarkable ability to sense, respond to, and adapt to their internal and external environments in order to maximize survival or accurately execute a developmental program. Such behavior requires the ability to process information, and cells have evolved complex regulatory and signaling systems capable of sophisticated information-processing tasks. It is ultimately the wiring of such systems and the relative quantitative strength of connections that confer on cells the ability to make decisions and regulate their behavior. Thus, there is a need to develop comprehensive, genome-wide maps of the complex signaling pathways operating inside cells. Although transcriptional networks in many organisms have recently been mapped on a global level using DNA microarrays, signaling pathways and networks can be considerably more difficult to study in a systematic, comprehensive fashion, requiring experimentally tractable systems amenable to a combination of genetic and biochemical methods.

**Figure 1:**
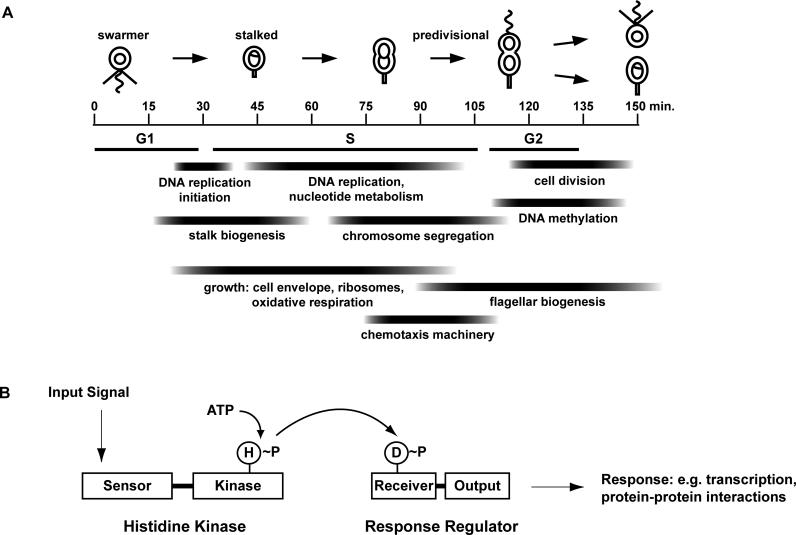
The *C. crescentus* Cell Cycle and Two-Component Signal Transduction (A) Schematic diagram of progression through the *C. crescentus* cell cycle, as described in the text. The timing of key cell cycle and developmental events are indicated. Cell division is asymmetric, generating two distinct daughter cells. The stalked cell can immediately initiate DNA replication, whereas the swarmer cell must first differentiate into a stalked cell. (B) Diagram of a canonical two-component signal transduction system. On receipt of an input signal, the histidine kinase autophosphorylates on a conserved histidine residue. The phosphoryl group is then passed to the receiver domain of a cognate response regulator. Phosphorylation of the receiver domain typically activates the output domain, which can execute a variety of cellular tasks including initiating programs of gene expression, catalyzing metabolic reactions, or modifying protein–protein interactions.

Here we report the design and use of a suite of tools for the rapid, systematic mapping of signaling networks responsible for regulating growth, cell cycle progression, and differentiation in the Gram-negative bacterium *Caulobacter crescentus*. This organism has emerged as an excellent model system for studying regulation of cell cycle progression and development owing to its dimorphic lifestyle (Figure 1A) [1–3]. Each cell division produces two different daughter cells: a stalked cell and a swarmer cell. The motile, chemotactic swarmer cell is unable to initiate DNA replication. In response to poorly understood environmental and internal cues, a swarmer cell differentiates into a stalked cell by losing its polar flagellum, chemotaxis machinery, and polar pili, followed by growth of a stalk. This motile-to-sessile transition is accompanied by increased rates of growth and protein synthesis [4]. This transition also coincides with DNA replication initiation and is thus a G1–S cell cycle transition. A single round of DNA replication ensues, followed by segregation of the daughter chromosomes to opposite ends of the predivisional cell. The development of the predivisional cell includes construction of a new flagellum, chemotaxis machinery, and pili secretion apparatus at the pole opposite the stalk. Cell division is asymmetric, producing a stalked cell that immediately reinitiates another round of DNA replication and a swarmer cell that will again differentiate into a stalked cell. Swarmer cells can be easily isolated from a mixed population of cells by density centrifugation and followed as they proceed synchronously through the cell cycle.

The regulation of this complex life cycle centers on a single class of signaling molecules known as two-component signal transduction systems. These systems are one of the key signaling modalities in the bacterial kingdom, as well as being present in fungi, slime molds, and plants [5]. As they appear to be absent from metazoans, including humans, this class of molecules has been suggested as a major new target for antibacterial and antifungal drug development [6,7]. The canonical two-component signal transduction system is shown in Figure 1B. A histidine kinase, often in response to receipt of a signal or stimulus, autophosphorylates on a conserved histidine residue. The phosphoryl group is then transferred to a conserved aspartate residue of a cognate response regulator. Phosphorylation of the response regulator occurs within the receiver domain and typically leads to a change in cellular physiology by activating an output domain. In many cases, phosphorylation enables the response regulator to bind DNA and function as a transcription factor. However, many other types of output domains are found that endow their response regulators with the ability to mediate protein–protein interactions or to perform enzymatic functions [8]. Two-component signaling pathways have been shown to respond to a wide range of stimuli, including sugars, peptides, antibiotics, and quorum-sensing signals. These signals trigger major physiological changes by changing programs of gene expression, altering swimming behavior, regulating proteolysis, or triggering differentiation [9,10].

Both histidine kinases and their targets, the response regulators, are easily identified in bacterial genomes solely by sequence homology. *C. crescentus* encodes 106 such proteins: 62 histidine kinases and 44 response regulators [11]. Some bacterial genomes encode as many as 250 of these signaling proteins, often amounting to more than 5% of all genes in a genome [12]. In *Escherichia coli,* the vast majority of two-component systems are encoded as operons of a histidine kinase and a response regulator that form an exclusive one-to-one phosphotransfer pair [13]. However, studies in *C. crescentus* and other bacteria reveal that two-component signaling pathways can often be highly branched, with many-to-one and one-to-many phosphotransfer relationships [5,14,15]. Such pathways are also often composed of kinases and regulators encoded in different operons scattered throughout a genome. In *C. crescentus* 41 histidine kinases and 19 response regulators, or 57% of all two-component genes, are orphans, not encoded in the same operon as another two-component gene. Identifying the connectivity of two-component signaling pathways is not possible by sequence analysis alone and is currently a major challenge. A recent report has attempted to map all such interactions in *E. coli* by systematically measuring phosphotransfer relationships between histidine kinases and response regulators [16].

Forward genetic screens in *C. crescentus* have identified 14 of the 106 two-component signaling genes as involved in cell cycle progression or differentiation (reviewed in [3,15]). The majority of these 14 are orphans and their connectivity remains poorly defined. Moreover, what role the other 92 two-component genes may play in regulating cell cycle progression and differentiation is largely unknown. Previous genetic screens may not have been saturated or may have had inherent biases, precluding identification of other important two-component regulators. To address these challenges, we undertook a systematic, comprehensive genetic and biochemical dissection of all 106 two-component signal transduction genes in the *C. crescentus* genome. Analysis of a complete set of deletion mutants identified 39 genes required for some aspect of growth or cell cycle progression, including nine essential genes. To identify phosphotransfer relationships, we developed a global in vitro biochemical approach that allows the identification of connections that are relevant in vivo. This technique takes advantage of data demonstrating that histidine kinases have an in vitro kinetic preference for their in vivo substrates. We demonstrate the utility of this integrated suite of systematic genetic and biochemical tools by identifying a previously unknown, but highly conserved, two-component pathway that is essential for growth of *C. crescentus* owing to a role in controlling cell envelope structure and integrity. The tools and approach presented can be applied to the study of two-component signaling proteins in other prokaryotes, including pathogens, and in any species having multiple two-component signaling systems, such as plants.

## Results

### Systematic Deletion of Two-Component Signaling Genes

**Figure 2:**
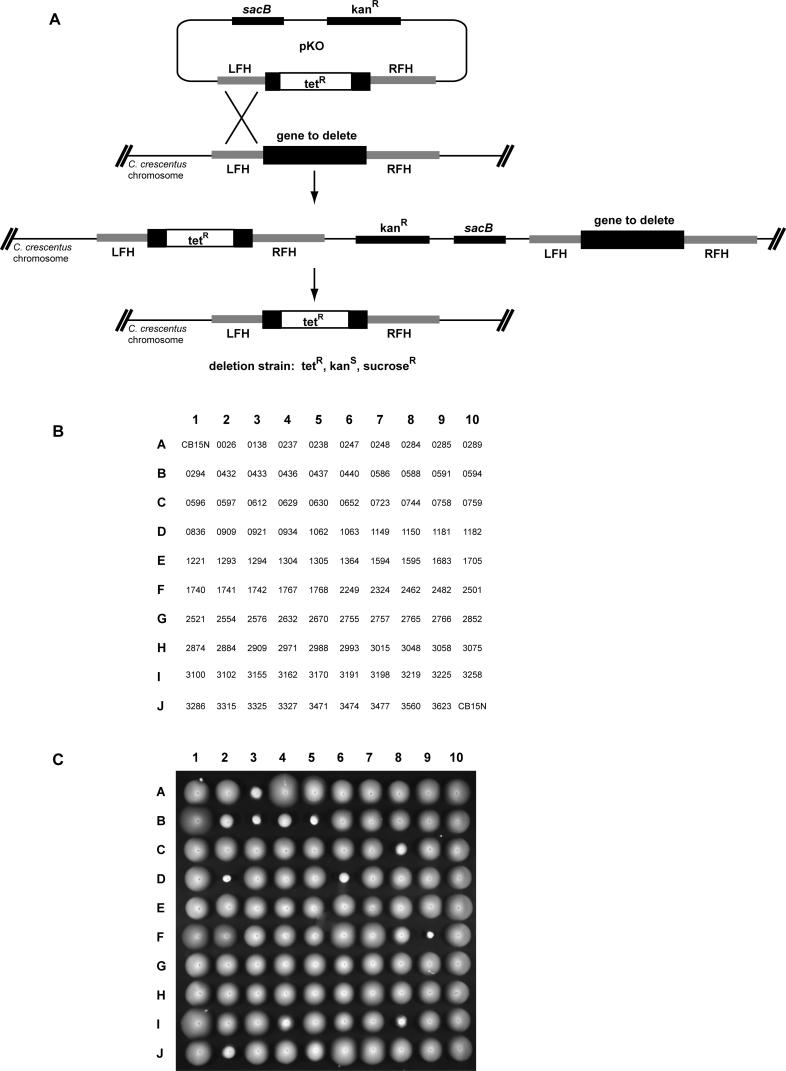
Systematic Deletion of Two-Component Signal Transduction Genes (A) Methodology used to generate chromosomal deletion strains. For each gene to be deleted, a suicide vector was constructed, with approximately 800-bp regions of homology upstream and downstream of the gene flanking a tet^R^ cassette. See Materials and Methods and Figure S1 for details of plasmid construction. In a two-step process, deletion strains are isolated by selecting first for tetracycline resistance and then by sucrose counter-selection utilizing the *sacB* gene carried on the vector. Cells harboring the *sacB* gene die in the presence of sucrose. Hence, a deletion strain is identified as tet^R^/sucrose^R^. For nonessential genes, stable deletions are easily identified by screening 5–10 colonies after the two-step recombination. For essential genes, no tet^R^/sucrose^R^ strains can be recovered (see text and Figure S1 for additional details). (B and C) Swarm plate analysis of 97 nonessential two-component deletion strains. (B) Map of strain positions in the swarm plates. Wild-type CB15N is in positions A1 and J10 for comparison to mutant strains. (C) PYE swarm plate after 3 d of growth at 30 °C. Swarm sizes and densities were scored visually and digital images analyzed in Matlab (MathWorks, Natick, Massachusetts, United States). Strains exhibiting swarm plate phenotypes are listed in Table 2, except for ΔCC1221 in position E1, which is deleted for a kinase erroneously annotated as a histidine kinase.

We analyzed the *C. crescentus* genome and identified 106 genes that encode members of the two-component signal transduction family: 62 histidine kinases and 44 response regulators (for annotation procedures, see Materials and Methods). To begin comprehensive identification of two-component signaling pathways required for cell cycle progression, cell growth, or cell polarity in *C. crescentus,* we generated deletion strains for each of the histidine kinase and response regulator genes identified. Deletions were made using long-flanking homology constructs carried on suicide vectors and a two-step recombination process (Figure 2; Materials and Methods). Selection for tetracycline resistance ensures integration of the suicide vector, and growth on sucrose (*sacB* is lethal when sucrose is present in the medium) selects for plasmid excision and formation of a stable deletion strain (Figures 2A and Figures S1). The two-step deletion procedure allows rapid identification of essential genes. If a gene is essential, the second recombination event always fails, and stable deletions (tetracycline-resistant [tet^R^]/sucrose-resistant [sucrose^R^] colonies) cannot be recovered (Figures 2A and Figures S1). In such cases, all sucrose^R^/tet^R^ colonies recovered are a result of *sacB* mutation, not loss of *sacB*.

**Table 2:**
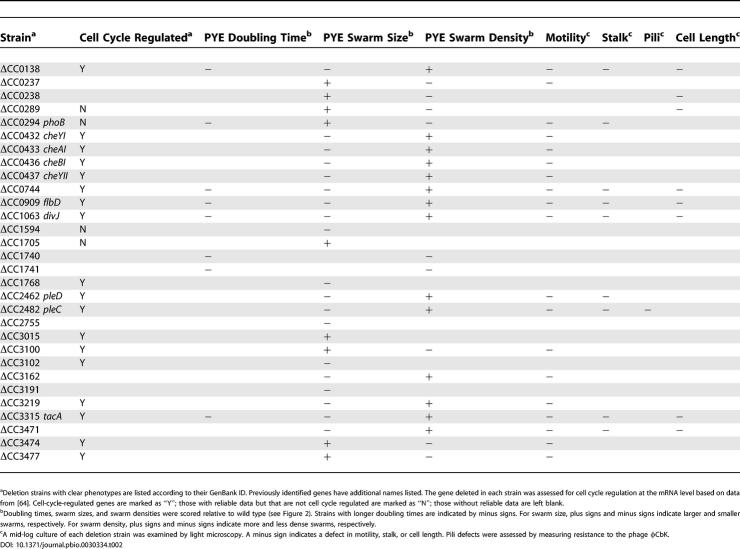
Nonessential Deletion Strains—Phenotypic Summary

We successfully generated stable deletion strains in rich medium (peptone yeast extract [PYE]) for 97 of the 106 *C. crescentus* two-component signaling genes. For these 97 genes, stable deletions were found after screening 5–10 colonies. For the remaining nine genes we tested at least 100 colonies after the final sucrose counter-selection (Figures 2A and Figures S1) and found that all still possessed the *sacB* gene, albeit inactivated. This suggests that each of these nine genes cannot be eliminated and hence each is essential for growth or viability (Table 1). This set includes all previously characterized essential two-component signal transduction genes in *C. crescentus: ctrA, cckA, divK,* and *divL* [17–21]. These results validate our method as a means to finding essential genes and strongly suggest that the five previously uncharacterized genes that could not be deleted (CC0530, CC1743, CC2931, CC2932, and CC3743) are also essential in *C. crescentus*.

**Table 1:**
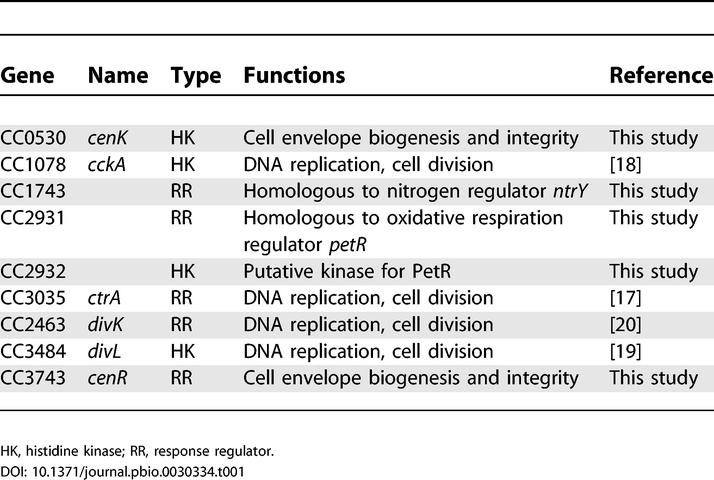
Essential Two-Component Signal Transduction Genes

CC0530 and CC3743 are both genes of unknown function. CC0530 encodes a predicted histidine kinase with two transmembrane domains and a periplasmic loop of about 130 amino acids. The protein encoded by CC3743 is a putative transcriptional regulator of the winged-helix OmpR subfamily (data not shown). CC2932 and CC2931 probably form an essential two-component pathway as orthologs of each are found in the same predicted operon, or adjacent operons, in a wide range of bacterial genomes. CC2931 encodes an ortholog of the response regulator PetR, which is essential in *Rhodobacter capsulatus* and required for oxidative respiration [22]. CC1743 is an ortholog of the gene *ntrY,* which may control growth in the presence of nitrate [23]. We thus suspected that CC1743 may be dispensable for growth in M2G minimal medium, where the sole nitrogen source is ammonium. Repeating the deletion procedure for CC1743 on minimal medium did in fact yield a stable deletion strain, so we classify CC1743 as a conditionally essential gene. We could not make any similar predictions for the other four new essential genes, and suggest that they are essential under most standard growth conditions.

### Phenotypic Analysis of Nonessential Deletion Strains

**Figure 3:**
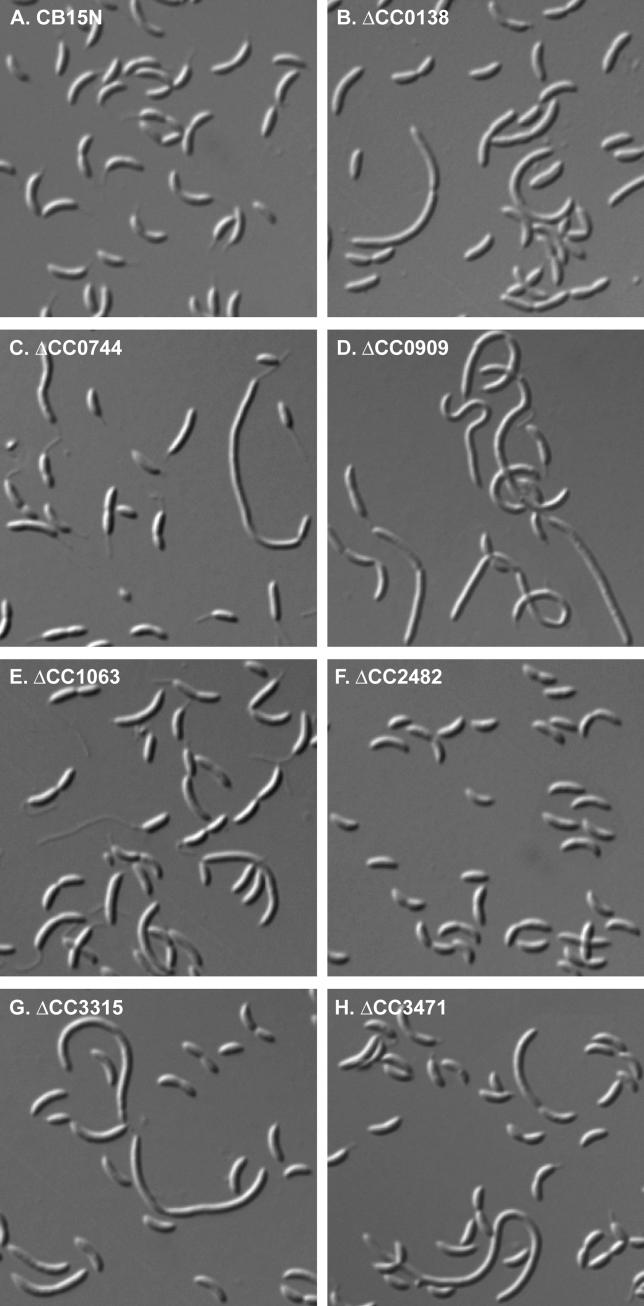
Morphology of Selected Deletion Strains Deletion strains were harvested at mid-log phase and imaged using differential interference contrast microscopy. Strains: (A) wild-type CB15N, (B) ΔCC0138, (C) ΔCC0744, (D) ΔCC0909, (E) ΔCC1063, (F) ΔCC2482, (G) ΔCC3315, and (H) ΔCC3471.

We next examined the phenotypes of the 97 nonessential deletion strains using a swarm plate assay. Wild-type cells can swim through low-percentage agar, creating a large, circular colony, or swarm, via the combined effects of chemotaxis and growth. Defects in a number of processes, including cell motility, chemotaxis, growth, cell division, and cell cycle progression, can produce changes in swarm size or density. The swarm plate assay is thus a rapid, sensitive, and comprehensive method for initial phenotypic characterization. Each deletion mutant, as well as the wild-type CB15N, was innoculated into swarm plates made from rich (PYE) medium, and swarms were photographed after three days (Figure 2B and 2C). From digital images, swarm size and swarm density were quantified for each deletion strain relative to wild-type (Figure 2C). Of the 97 deletion strains, 30 exhibited a significantly altered swarm size or density (Table 2). Each of these genes was further characterized by measuring the log-phase generation time in rich medium and by examining cellular morphology for abnormalities in cell shape, cell length, motility, and stalk formation (Figure 3; Table 2).

Strong candidates for cell cycle or cell growth regulatory genes are those marked by deletion strains that show a decrease in swarm size and a longer generation time. Five strains matching these criteria were found, including deletions of known cell cycle regulators *(divJ* and *flbD)* and three uncharacterized regulators (*tacA*, CC0138, and CC0744). Strains with smaller swarms but no change in generation time likely indicate genes required for motility or chemotaxis, and this group of genes includes the known chemotaxis *(che)* genes. Seven strains had larger and less dense swarms than wild-type, perhaps because of disruption of genes controlling the swarmer-to-stalked cell transition, leading to extended swimming.

In sum, the initial phenotypic characterization of our comprehensive library of two-component deletion strains has identified 39 genes (30 nonessential and nine essential)—or more than 35% of all two-component signaling genes—required for some aspect of growth, viability, morphogenesis, or cell cycle progression. This includes all 14 of the genes found by previous forward genetic screens for morphogenetic and cell cycle mutants (Tables 1 and 2), as well as 25 previously uncharacterized two-component signaling genes involved in regulating the *C. crescentus* life cycle. The uncharacterized genes are not simply those with subtle mutant phenotypes, as many have severe defects, including five that appear to be essential for growth or viability. Detailed characterization will be necessary to pinpoint the precise function of each of these uncharacterized genes.

### Systematic Biochemical Analysis of Two-Component Signal Transduction

**Figure 4:**
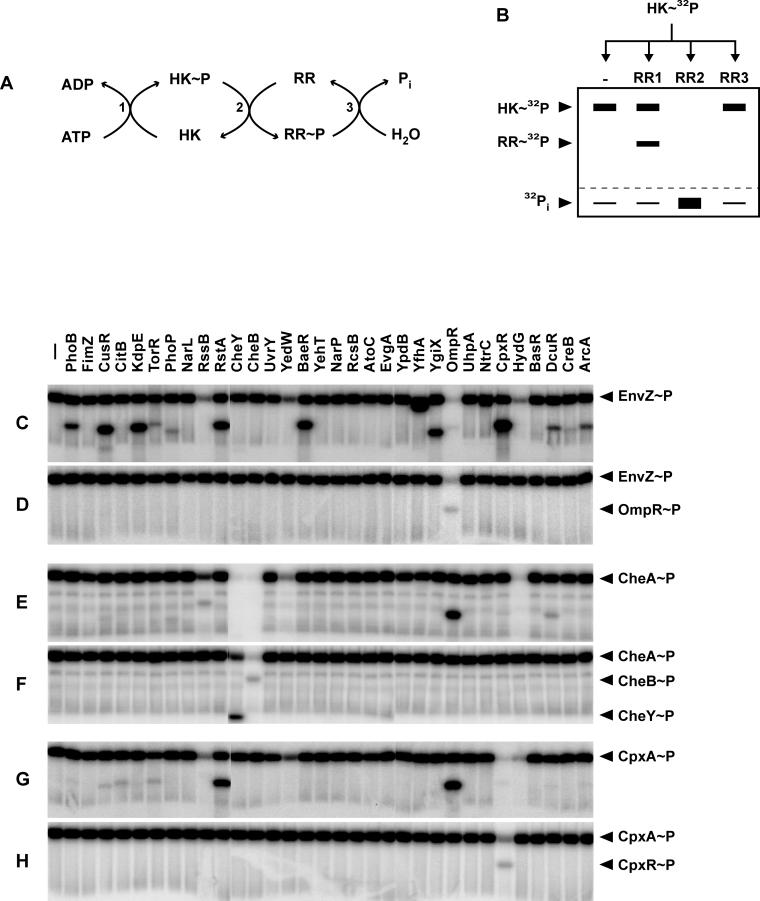
Phosphotransfer Profiling Method (A) Phosphotransfer profile experiments involve three separate reactions: (1) autophosphorylation of the histidine kinase (HK) by radiolabeled ATP, (2) phosphotransfer to a response regulator (RR), and (3) dephosphorylation of the response regulator. (B) Schematic of the phosphotransfer profiling technique. A single preparation of purified, autophosphorylated kinase (HK∼^32^P) is mixed with each response regulator from a given organism and analyzed for phosphotransfer by SDS-PAGE and autoradiography. The first lane shows a single band corresponding to the autophosphorylated histidine kinase and is used as a comparison for every other lane. Lanes 2–4 illustrate the three possible outcomes of a phosphotransfer reaction. In lane 2, phosphotransfer from HK to RR1 leads to the appearance of a band corresponding to RR1. In lane 3, phosphotransfer from HK to RR2 also occurs, but owing to high phosphatase activity (either autophosphatase or catalyzed by a bifunctional HK), the net result is production of inorganic phosphate (Pi) and the depletion of radiolabel from both the HK and RR2. In lane 4, no phosphotransfer occurs, and the lane is indistinguishable from lane 1. (C–H) Phosphotransfer profiling was performed for three *E. coli* kinases (EnvZ, CheA, and CpxA) against all 32 purified *E. coli* response regulators, with phosphotransfer incubation times of either 1 h (C, E, and G) or 10 s (D, F, and H). For these three histidine kinases, a comparison of the short and long time point profiles indicates a kinetic preference for only their in vivo cognate regulators: OmpR (C and D), CheY and CheB (E and F), and CpxR (G and H). After being examined for phosphotransfer, all gels are stained with Coomassie to verify equal loading of histidine kinase and response regulator in each lane (data not shown). For each kinase profiled, we purified only its soluble, cytoplasmic domain, either as a thioredoxin-His_6_ or a His_6_-MBP fusion, using standard metal affinity chromatography (see Materials and Methods). When necessary, we made successive N-terminal truncations until we identified a construct that produced active kinase in vitro, always preserving the H-box and ATP binding domain (details on constructs used are in Table S3). All response regulators were purified as full-length fusions to a thioredoxin-His_6_ tag. Purity was assessed by Coomassie staining, with each purified kinase domain and response regulator, except for *E. coli* FimZ, yielding an intense band of the correct approximate molecular weight (see Figure S5; Table S3).

As a first step in further characterization of the two-component signaling genes involved in the cell cycle progression and development of *C. crescentus,* we sought to identify the response regulator targets of each histidine kinase. For orphan kinases and regulators, cognate pairs cannot easily be predicted based on sequence analysis alone. Of the 39 mutants showing phenotypes in the assays described above, 26 are orphans and their phosphotransfer pairings thus unknown. To systematically identify connectivity between two-component signaling proteins, we developed a global in vitro biochemical technique, which we term phosphotransfer profiling, to rapidly identify the targets of histidine kinases (Figure 4).

In a profiling experiment (Figure 4A and 4B), the purified cytoplasmic, soluble kinase domain of a histidine kinase is autophosphorylated with [γ-^32^P]ATP, and then split into separate reactions containing equimolar amounts of each purified, full-length response regulator (for details of protein purification, see Materials and Methods). Each phosphotransfer reaction is incubated for an identical period of time and then stopped by addition of sample buffer, separated by SDS-PAGE, and imaged on phosphor screens. A control of autophosphorylated kinase without any added response regulator is included for reference, and forms a single intense band. Efficient phosphotransfer to a response regulator can be manifested in two ways (Figure 4B). In the first case, a high-intensity band is seen at the appropriate molecular weight for phosphorylated response regulator. In the second case, efficient phosphotransfer can lead to depletion of radiolabel from the histidine kinase band. As some response regulators have high autophosphatase activity and some histidine kinases are bifunctional, also acting as specific phosphatases for their cognate response regulators, the net result of efficient phosphotransfer and phosphatase activities is the depletion of radiolabel from the autophosphorylated kinase (Figure 4A and 4B) [24]. Hence, to identify a phosphotransfer relationship, each reaction in a profile assay is inspected for (i) a band corresponding to the response regulator or (ii) a decrease in intensity of the kinase band relative to the kinase-only control. Importantly, because our profile method relies on the comparison, in parallel, of all potential phosphotransfer substrates for a given kinase, it is independent of the specific activity of the kinase being tested.

### Histidine Kinases Exhibit a System-Wide In Vitro Kinetic Preference for Their Cognate Response Regulators

We chose to test and validate our in vitro profiling technique using purified kinases and response regulators from *E. coli* as many of its in vivo phosphotransfer pairings are known. First, we characterized phosphotransfer to response regulators by the histidine kinase EnvZ, which responds in vivo to changes in osmolarity by controlling the phosphorylation state of the response regulator OmpR [25,26]. The profile of EnvZ after a 1-h reaction time with each of the 32 purified *E. coli* response regulators demonstrates phosphotransfer to 11 different response regulators, including OmpR (Figure 4C). However, with a shorter, 10-s reaction time the only efficient phosphotransfer is to OmpR (Figure 4D), demonstrating a clear kinetic preference of EnvZ for its cognate substrate OmpR. We next tested the CheA histidine kinase, which phosphorylates CheY and CheB in vivo to control chemotaxis [27,28]. At 1 h, CheA shows phosphotransfer to seven response regulators, including CheY and CheB (Figure 4E), but at 10 s we detect only phosphorylation of CheY and CheB (Figure 4F). We then tested a third kinase, CpxA, which is known to signal through CpxR in vivo [29]. With the long reaction time, CpxA phosphorylates CpxR as well as several other response regulators (Figure 4G). The short reaction time again reveals a kinetic preference of the kinase CpxA for its in vivo, cognate substrate, CpxR (Figure 4H). We have observed similar kinetic preferences of two other *E. coli* kinases, PhoQ and PhoR, for their respective phosphotransfer substrates, PhoP and PhoB (data not shown). We conclude that *E. coli* histidine kinases have a strong kinetic preference for their in vivo cognate response regulators, with promiscuity only observed after extended incubation times. We have estimated the kinetic preference of kinases to be at least 10^3^ in terms of relative *k*_cat_/*K*_m_ ratios (Figure S2).

**Figure 5:**
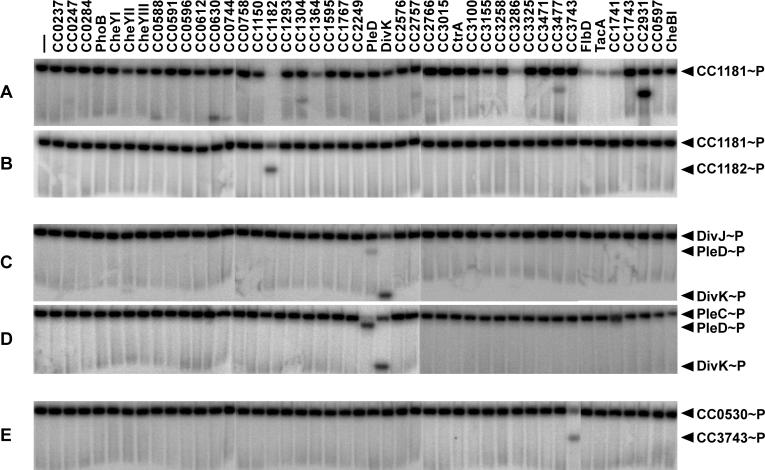
Phosphotransfer Profiling of *C. crescentus* Histidine Kinases Profiles for four purified *C. crescentus* kinases versus 44 purified response regulators were obtained by the method described for *E. coli* in Figure 4. (A) One-hour time point profile of the *C. crescentus* kinase CC1181. (B) Ten-second time point profile. Only CC1182, encoded in the same operon as CC1181 and the likely in vivo target, is phosphorylated at the short time point. Kinetic preference of *C. crescentus* histidine kinases for their cognate substrates was similarly demonstrated for five other operon pairs (data not shown). (C and D) Ten-second time point profiles of the orphan kinases DivJ and PleC, demonstrating phosphorylation of only their shared in vivo targets, PleD and DivK. (E) Phosphotransfer profiling of the previously uncharacterized essential orphan kinase CC0530 (CenK) reveals a single preferred substrate, CC3743 (CenR).

Next, we tested *C. crescentus* histidine kinases to determine if kinetic preference for substrates extends to the two-component systems in this organism. We started by profiling a two-component pair of unknown function: CC1181/CC1182. Because the kinase and regulator are encoded in the same operon they likely form an exclusive phosphotransfer pair in vivo. As with *E. coli* histidine kinases, we found that multiple response regulators were phosphorylated by CC1181 at the 1-h time point, including CC1182 (Figure 5A). However, a shorter phosphotransfer incubation time of 10 s revealed a clear kinetic preference of CC1181 for CC1182 (Figure 5B). We then tested five other *C. crescentus* histidine kinases, CC0289 (PhoR), CC0759, CC1740, CC2765, and CC3327. In each case, the histidine kinase exhibited a strong kinetic preference for its known substrate or the substrate encoded within its own operon, CC0294 (PhoB), CC0758, CC1741, CC2766, and CC3325, respectively (data not shown).

Next, we used profiling with orphan *C. crescentus* histidine kinases for which the cognate response regulators could not be predicted by sequence analysis alone. First, we tested the orphan kinases DivJ and PleC, which were identified in our deletion analysis, and in previous genetic screens [30,31], to be key regulators of cell cycle progression and morphogenesis. Both of these kinases have been shown previously to phosphorylate the essential response regulator DivK and the response regulator PleD, which are in the same operon together, but without an adjacent kinase [20,21,32]. Short, 10-s reaction time profiles of DivJ and PleC demonstrate a kinetic preference for DivK and PleD and suggest that these are the exclusive targets of DivJ and PleC (Figure 5C and 5D).

We conclude that, as in *E. coli, C. crescentus* histidine kinases have an in vitro kinetic preference for their in vivo cognate substrate. Kinetic preference of a kinase for its cognate response regulator has been noted before on a limited scale [25,33–35], but our data extend this observation to a genome-wide level. Moreover, we suggest that this kinetic preference can be exploited to rapidly identify in vivo phosphotransfer relationships.

### Identification of a New Essential Two-Component Pathway That Controls Cell Envelope Integrity

The systematic deletion analysis described above identified four histidine kinases that each appear to be essential for growth or viability: *divL, cckA,* CC2932, and CC0530 (see Table 1). *divL* and *cckA* have both been previously identified as essential regulators and are implicated in phosphorylation of the essential response regulator CtrA [18,19]. CC2932 is encoded in an operon with the essential response regulator CC2931, and these probably form a phosphotransfer pair. CC0530, however, is a previously uncharacterized, orphan kinase with no known or predicted substrate. Using phosphotransfer profiling, we demonstrated that CC0530 preferentially phosphorylates a single target, the orphan response regulator CC3743 (Figure 5E). As with CC0530, we had identified CC3743 as a previously uncharacterized orphan gene that is likely essential for growth or viability of *C. crescentus* (see Table 1). Together our genetic and biochemical observations strongly suggest that these two orphans comprise an essential two-component pathway in *C. crescentus*.

To test whether CC0530 and CC3743 are indeed essential, we generated strains in which the only copy of each gene is present on a low-copy plasmid under the control of the xylose-inducible, glucose-repressible promoter P*_xylX_*. For both genes, stable deletions were easily recovered when these complementing plasmids were present but not in the presence of an empty vector control (Table 3). This work produced strain ML521 (ΔCC0530 + P*_xylX_*-CC0530) and strain ML550 (ΔCC3743 + P*_xylX_*-CC3743). ML521 formed colonies only on plates supplemented with xylose, consistent with the CC0530 histidine kinase being essential for growth (data not shown). In contrast, ML550 formed colonies in the presence of xylose or glucose. We suspected that CC3743 may be a stable protein and hence difficult to deplete when expressed from a plasmid. We therefore made a destabilized version of CC3743 by adding a C-terminal *ssrA* tag, which targets proteins for degradation and decreases protein half-life inside the cell [36]. Using this destabilizing tag, we successfully created the strain ML591 (ΔCC3743 + P*_xylX_*-CC3743-*ssrA*), which forms colonies on PYE plates supplemented with xylose but not with glucose (data not shown). The ability of ML591 to grow on medium with xylose suggests that the *ssrA* tag does not interfere with the function of CC3743, but does allow the depletion of CC3743 during growth on glucose. The depletion strains ML521 and ML591 also grew only in minimal medium supplemented with xylose (M2X) and not with glucose (M2G), supporting the general essential nature of these two genes (data not shown).

**Table 3:**
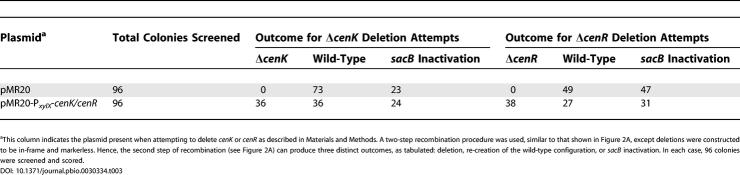
CC0530 *(cenK)* and CC3743 *(cenR)* Are Essential Genes

**Figure 6:**
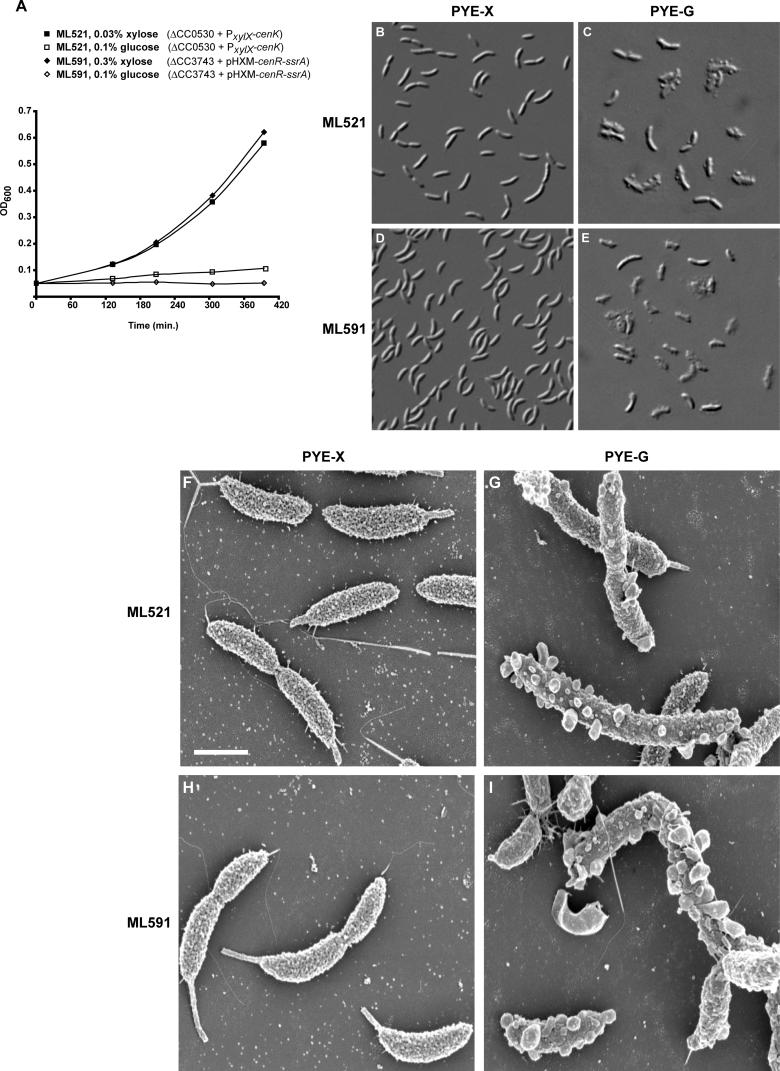
CC0530 *(cenK)* and CC3743 *(cenR)* Are Essential for Growth and Required for Cell Envelope Integrity Growth curves for the ML521 (ΔCC0530 + P*_xylX_*-*cenK*) and ML591 (ΔCC3743 + pHXM-*cenR-ssrA*) depletion strains (A). Overnight cultures of each were grown in PYE plus xylose (PYE-X), washed with plain PYE, and diluted in PYE plus xylose or PYE plus glucose (PYE-G). After 12 h of growth in these conditions cells reached an optical density (OD_600_) level that could be measured (this time is plotted as “0 min”). Morphology was observed by light microscopy for the *cenK* depletion (ML521) after a total of 20 h in PYE plus xylose (B) or PYE plus glucose (C) and for the *cenR* depletion (ML591) after 20 h in PYE plus xylose (D) or PYE plus glucose (E). Scanning electron micrographs under identical conditions are shown for ML521 in PYE plus xylose (F) and PYE plus glucose (G) and for ML591 in PYE plus xylose (H) and PYE plus glucose (I). For (F–I), scale bar represents 1 μm. Depletion of either gene product led to an unusual, irregular blebbing of the cell surface. Cells were not motile, and had reduced stalk length.

Next, we examined the phenotype of these strains in liquid medium after depleting each gene product. Cultures of each were grown in rich medium supplemented with xylose and then washed and resuspended at a low density in medium with xylose or glucose. We measured the growth rate and observed the cells by light microscopy (Figure 6A–6E). In the presence of xylose, growth of ML521 and ML591 was virtually indistinguishable from wild-type, suggesting that expression of either CC0530 or CC3743 under these conditions has no deleterious effect (Figure 6A). However, when shifted to glucose, the cultures of each depletion strain stopped growing and failed to accumulate significant optical density (Figure 6A). After ∼20 h of depletion by growth in glucose, we examined the morphological phenotype of each strain by light microscopy. Depletion of either gene product led to loss of motility, shorter stalks, and a dramatic, unusual membrane blebbing, resulting in bubble-like protrusions on the cell surface (Figure 6C and 6E). Cells were approximately wild-type in length and size, but had cell envelope blebs nearly covering the cell surface. We reasoned that the blebs were contiguous extrusions of the cell envelope that did not disrupt permeability as these cells did not lyse even after extended incubation in glucose-containing medium. Using high-resolution scanning electron microscopy, we examined cells from each depletion strain after extended growth in xylose and glucose. Consistent with the light microscopy results, we observed large, irregular protrusions across the surface of the cells grown in glucose and depleted of CC0530 or CC3743 (Figure 6F–6I). The growth and morphological phenotypes of the two depletion strains were nearly identical, further supporting the conclusion that CC0530 and CC3743 participate in the same signal transduction pathway. Based on our observations we have named CC0530 and CC3743 *cenK* (cell envelope kinase) and *cenR* (cell envelope regulator), respectively.

**Figure 7:**
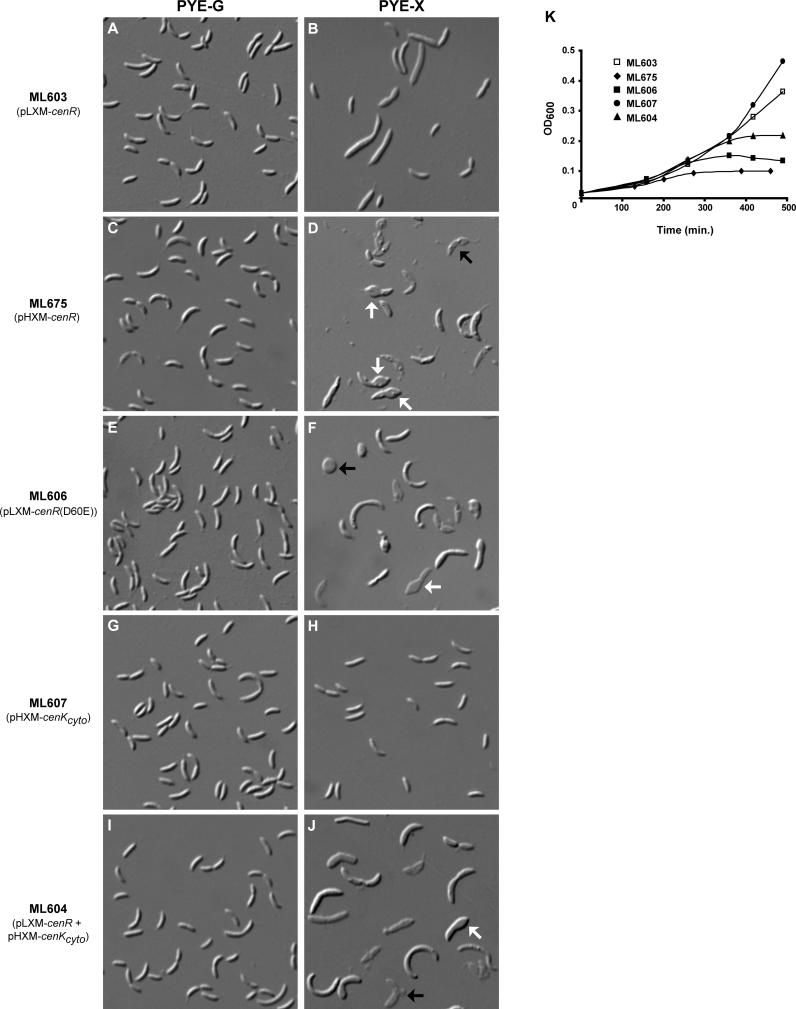
Constitutive Activation of the CenK–CenR Pathway Leads to Dramatic Changes in Cell Morphology, Cell Lysis, and Death Images are shown for strains grown overnight in PYE plus glucose and then diluted back to early log phase and grown for 5 h in PYE plus glucose (A, C, E, G, and I) or xylose (B, D, F, H, and J). In all panels, white arrows indicate cells with asymmetric bloating and black arrows indicate lysed cells. (A and B) ML603 (CB15N + pLXM-*cenR* + pJS71) expresses CenR alone from a low-copy vector. (C and D) ML675 (CB15N + pHXM-*cenR*) expresses CenR alone from a high-copy vector. (E and F) ML606 (CB15N + pLXM-*cenR*[D60E]) expresses, from a low-copy vector, a mutant of CenR that mimics constitutive phosphorylation. (G and H) ML607 (CB15N + pMR20 + pHXM-*cenK*_cyto_) expresses CenK_cyto_ alone from a high-copy vector. (I and J) ML604 (CB15N + pLXM-*cenR* + pHXM-*cenK*_cyto_) expresses both CenR and CenK_cyto_, from low- and high-copy plasmids, respectively. (K) Growth curve for all strains from (A–J) grown in PYE supplemented with xylose [64,65].

To understand the functions of the *cenK–cenR* pathway in more detail we examined the effects of overexpressing components of this pathway (Figure 7). First we examined the phenotype of strain ML603, which expresses a full-length copy of *cenR* under control of the P*_xylX_* promoter on a low-copy plasmid (pMR20) in a wild-type background. In the presence of glucose, cells of this strain were virtually indistinguishable from wild-type cells (Figure 7A). However, in the presence of xylose, these cells showed significant cellular elongation, and many cells appeared to be losing their shape, exhibiting a bloated, enlarged morphology (Figure 7B). To increase expression further, we constructed a strain (ML675) with P*_xylX_*-*cenR* on pJS71, a higher-copy-number vector than pMR20. In the presence of glucose, strain ML675 also appeared similar to wild-type (Figure 7C), but growth in xylose revealed a dramatic morphological phenotype, ranging from bloated, enlarged cells to pervasive cell lysis (Figure 7D). Measurements of optical density after shift to xylose indicated a rapid growth arrest (Figure 7K). Interestingly, we noted that in many predivisional cells, the cell was enlarged asymmetrically, always with the stalked half of the cell losing its rod-like appearance (indicated by white arrows in Figure 7D). These data, together with the depletion analysis, suggest that *cenR* is involved in maintaining proper cell envelope structure, and further suggest that peptidoglycan or cell membrane synthesis may proceed in an asymmetric fashion in wild-type *C. crescentus* cells.

For many response regulators, mutating the conserved phosphorylation site from aspartate to glutamate mimics constitutive phosphorylation [37,38]. We introduced such a mutation, D60E, into *cenR,* on a low-copy plasmid. In the presence of glucose, the resulting cells looked similar to wild-type (Figure 7E), but when shifted to xylose, they became severely enlarged, lost their usual rod shape, and within 5 h began to lyse and die (Figure 7F and 7K). Thus, the phenotype of overexpressing CenR(D60E) on a low-copy plasmid matched that of overexpressing wild-type CenR on a high-copy plasmid (compare Figure 7D and 7F). We conclude that the D60E mutation leads to phosphorylation-independent activity of CenR. We also attempted to generate strains expressing CenR(D60E) from the high-copy plasmid pJS71, but no colonies were recovered, even on glucose plates, suggesting that the D60E allele may be so active that even basal expression in glucose is lethal.

Unlike with CenR, overexpression of the full-length CenK (data not shown) or its cytoplasmic kinase domain had no effect on cell growth or cell morphology (Figure 7G and 7H). This may be because the amount of CenR is limiting in the cell, so that additional CenK expression may not alter the fraction of phosphorylated CenR. Alternatively, the cell may be robust to changes in kinase concentration, as suggested for the kinase EnvZ [39]. Regardless, we predicted that if CenK is the in vivo cognate kinase for CenR, then simultaneously overexpressing both CenK and CenR should phenocopy overexpression of CenR(D60E). As expected, the effect of co-overexpressing CenK_cyto_ and CenR (Figure 7J) was significantly more severe than that of expressing either protein alone (compare to Figure 7B and 7H), and phenocopied the overexpression of CenR(D60E) (Figure 7F). As a control to ensure that the effect was due to kinase activity of CenK, we mutated the active-site histidine to alanine (H273A) and showed that the growth rate of cells co-overexpressing CenK(H273A) and CenR was nearly indistinguishable from that of cells overexpressing CenR alone (data not shown). These results support the conclusion that CenK acts in vivo to phosphorylate, and hence activate, CenR, as suggested by the in vitro phosphotransfer profiling.

The CenK–CenR pathway appears to be widely conserved throughout the alpha subdivision of proteobacteria. Multiple sequence alignments indicate better than 60% identity (70% similarity) for CenR and better than 35% identity (50% similarity) for CenK (Figures S3 and S4). The similarity extends throughout the full length of each protein, including the putative periplasmic ligand-binding domain of CenK. We suggest that the CenK–CenR pathway may be essential and function similarly in a range of other bacteria.

## Discussion

### Systematic Deletion of Two-Component Signal Transduction Genes

By deleting each of the 106 two-component signal transduction genes encoded in the *C. crescentus* genome, we have identified 39 mutant strains with cell cycle or developmental phenotypes (see Tables 1 and 2). Previous forward genetic screens had identified 14 two-component signaling genes involved in cell cycle progression and morphogenesis in *C. crescentus,* including four essential for viability of the organism. However, forward genetic screens are typically designed to select for a particular phenotype or may not be screened to saturation. The comprehensive, unbiased nature of the reverse genetic approach taken here expands both the number and role of two-component signaling proteins in regulating the *C. crescentus* cell cycle. The newly identified mutants include many with severe phenotypes as well as four previously uncharacterized genes that appear to be essential for growth or viability in both rich and minimal media. The library of deletion strains created here will also serve as a resource for future explorations of two-component regulation in *C. crescentus*. The deletion strains can be individually characterized in more depth, in different conditions, or even in different strain backgrounds. In addition, the inclusion of unique molecular bar codes in each strain (see Materials and Methods) opens the possibility of parallel fitness studies similar to those used for the *Saccharomyces cerevisiae* whole-genome deletion collection [40,41].

### Systematic Biochemical Analysis of Two-Component Phosphorylation

Similarity of mutant phenotypes can help to identify two-component genes acting in the same pathway, but ultimately, a biochemical demonstration of phosphorylation is required to define signal transduction pathways. Such a combination of genetics and biochemistry has successfully defined individual two-component signaling pathways in a number of organisms [10], but this report presents a global, integrated genetic and biochemical study of a bacterium's complete set of two-component signal transduction systems.

Histidine kinases have been widely thought to function promiscuously in vitro, precluding correspondence with in vivo targets. However, a few studies have suggested that histidine kinases may have a kinetic preference in vitro for their in vivo cognate substrates. For example, in *Bacillus subtilis,* the kinase KinA can phosphorylate both Spo0A and Spo0F in vitro, but has a more than 50,000-fold preference, as measured by relative *k*_cat_/*K*_m_ ratios, for Spo0F, its in vivo cognate substrate [35]. A similar magnitude of kinetic preference was shown for the kinase VanS phosphorylating its cognate regulator VanR relative to the noncognate substrate PhoB [34]. The phosphotransfer profiling data presented here extend these observations to a system-wide level and suggest that the apparent promiscuity of histidine kinases in vitro is attributable to excessive incubation times or a high concentration of reaction components, each of which acts to cross the kinetic barrier that enables a kinase to selectively phosphotransfer to its cognate substrate. A recent comprehensive study of two-component signal transduction in *E. coli* examined phosphotransfer in vitro from each histidine kinase to each response regulator at a 30-s time point [16]. As with our data, all known cognate pairs showed significant phosphotransfer, but the study reported a small number of interactions between noncognate pairs [16]. However, the in vivo relevance of these interactions is not yet known, and because that study did not examine phosphotransfer at multiple time points, the strength of noncognate interactions relative to those of cognate pairs is also not yet clear.

Our profiling method examines, simultaneously and in parallel, the ability of a purified histidine kinase to phosphorylate each of the response regulators encoded in that organism's genome. It would be impractical to determine *k*_cat_/*K*_m_ for each kinase–regulator combination, but kinetic preference can still easily be seen by conducting comprehensive profiles at multiple time points. Importantly, using a number of previously well-characterized *E. coli* histidine kinases, we demonstrated a direct correspondence between this kinetic preference and in-vivo-relevant response regulator substrates (see Figure 4). We were then able to use this kinetic preference to identify in vivo targets of uncharacterized histidine kinases such as the *C. crescentus* orphan CenK (see Figure 5E). Note, however, that phosphotransfer profiling is not used in isolation to identify phosphotransfer pairs, but is integrated with genetic data and in vivo experiments, as demonstrated here for CenK–CenR.

The phosphotransfer profiling technique is robust to a number of experimental variables. First, it is independent of the specific activity of the purified histidine kinase, because the method relies on a relative comparison of phosphotransfer kinetics from a single preparation of kinase to each possible substrate. Second, because the kinetic preference of kinases appears to be on the order of 10^3^ or even 10^4^, the method is not significantly affected by differences in response regulator concentration, even differences as great as 10-fold. Also, some histidine kinases are bifunctional, acting as both a kinase and a phosphatase for their cognate response regulators. In most cases, control of the relative ratio is not understood in vivo, making it difficult to predict the ratio of kinase to phosphatase activity of a particular purified construct in vitro. Any construct having net kinase activity can be profiled by our method to identify the probable in vivo substrates, but determining whether the histidine kinase acts predominantly as a kinase or a phosphatase in vivo depends on integration with genetic and other in vivo observations. For example, our profiles of DivJ and PleC, as well as previous studies of these kinases, suggest that both target the regulators DivK and PleD [20,21,32]. In vivo, though, DivJ is thought to function primarily as a kinase for DivK and PleD, whereas the bifunctional kinase PleC appears to act as a phosphatase [42,43].

### Identifying Novel Signal Transduction Systems

We demonstrated the integration of our genetic and biochemical methods to identify a novel, essential pathway from the histidine kinase CenK to the response regulator CenR, which appears to control critical aspects of cell envelope integrity. CenK is a predicted transmembrane protein with a periplasmic domain of ∼130 amino acids, although no periplasmic stimulus could be predicted based on sequence. CenR is a predicted DNA-binding protein of the OmpR subfamily, so defining the CenR regulon may help to unveil its role in controlling the cell envelope. Depletion of either gene product led to a severe membrane blebbing phenotype, which, to the best of our knowledge, has not been seen before in *C. crescentus*. A number of other *C. crescentus* genes are involved in maintaining cell wall integrity and cell shape, including *mreB, rodA,* and *cicA,* but the relationship, if any, of these genes to *cenK* and *cenR* is not yet clear [44–46].

CenK–CenR is, to our knowledge, the first essential two-component pathway discovered in Gram-negative bacteria controlling cell envelope processes. In some Gram-positive bacteria, an essential two-component pathway, YycG–YycF, also plays a role in cell envelope biogenesis [47–49], but does not appear to be orthologous to the CenK–CenR system. However, the CenK–CenR pathway does appear to be highly conserved throughout the alpha subdivision of proteobacteria, including a number of important plant, animal, and human pathogens. Two-component systems have been highlighted as a possible new antibiotic target given their absence in humans and other animals [6,7,50,51]. Furthermore, as the physical construction of the cell wall has long been a major target of antibiotics, the CenK–CenR regulatory pathway may be a particularly suitable target for novel antibiotic development.

### Signaling Pathway Specificity and Insulation

All organisms use a relatively small number of signaling modalities. For bacteria such as *C. crescentus* two-component signaling systems are widely employed, whereas eukaryotes have large families of other signaling systems, such as MAP kinase cascades, TGF-β pathways, and receptor tyrosine kinases. By definition, cross-talk between pathways must be minimal, otherwise an organism would be unable to trigger specific responses to specific stimuli. However, the mechanisms and strategies employed by cells to insulate highly related pathways are poorly understood and have been a recent focus of attention in many organisms [52–55].

We propose that the system-wide kinetic preference of histidine kinases for their cognate response regulators is a fundamental mechanism by which bacterial cells maintain the insulation of two-component signaling pathways. The large kinetic preference of kinases for their cognate substrates suggests that cross-talk observed in vitro likely arises from excesses in reaction time or reaction components and does not occur in vivo. Importantly, we distinguish deleterious cross-talk from cross-regulation in which a single kinase has multiple bona fide targets or multiple kinases regulate the same response regulator. There are several well-studied examples of cross-regulation, such as the *E. coli* kinase CheA, which phosphorylates both CheY and CheB as part of its role in regulating chemotaxis [27], and some of the noncognate interactions found in a systematic study of *E. coli* two-component signaling may represent additional cases of cross-regulation [16]. In *C. crescentus,* cross-regulation occurs between the orphan kinases DivJ and PleC, and the two response regulators DivK and PleD. Our profile data for CheA, DivJ, and PleC demonstrated that kinases involved in cross-regulation have approximately equal kinetic preference for their multiple response regulator targets (see Figure 5C and 5D).

There are, of course, many additional means by which cells ensure signaling specificity. For example, subcellular localization of interacting components, scaffolding, and mutual inhibition can all act to ensure specificity [53]. However, our in vitro results point to biochemical selectivity as a fundamental mechanism, on which other layers of regulation and insulation may be built. Recent results with the cyclin-dependent kinases suggest that biochemical selectivity may also play a fundamental role in this process in *S. cerevisiae* [54]. It remains a major challenge to understand in complete detail how organisms robustly and accurately ensure signal fidelity within a cell [55].

### Concluding Remarks

The techniques and approach described here can be directly extended to any organism containing two-component signal transduction systems, and are particularly useful for species with large sets of these molecules. This includes most bacteria, which typically encode at least 20 or 30 two-component genes and sometimes more than 100. Many plant species, including the model system *Arabidopsis thaliana* and the agriculturally and economically important rice plant *Oryza sativa,* also contain large sets of two-component signaling genes.

Finally, all cells, even relatively simple bacteria, are capable of complex information-processing tasks, such as converting continuous signals to discrete outputs, signal amplification, coincidence detection, and cellular-level memory. The successful implementation of these tasks is not carried out by individual proteins, but rather by multiple proteins, arranged into complex, highly connected circuits. For example, MAP kinase pathways are capable of converting continuous signals to an all-or-none output owing to a precise connectivity, a three-tiered MAPK cascade, and positive feedback [56]. Mapping the structure of signaling pathways and networks, as initiated here for *C. crescentus,* will thus be critical to our understanding of how cells process information and make decisions in order to regulate their behavior.

## Materials and Methods

### Bacterial strains, plasmids, and growth conditions

*E. coli* strains were routinely grown in Luria Broth (BD Biosciences, Franklin Lakes, New Jersey, United States) at 37 °C, supplemented with carbenicillin (100 μg ml^−1^ or 50 μg ml^−1^), chloramphenicol (30 μg ml^−1^ or 20 μg ml^−1^), kanamycin (50 μg ml^−1^ or 30 μg ml^−1^), oxytetracycline (12 μg ml^−1^), or spectinomycin (50 μg ml^−1^) as needed for solid and liquid media. *C. crescentus* strains were grown in PYE (complex medium) or M2G (minimal medium) at 30 °C [57]. PYE medium was supplemented with 3% sucrose, oxytetracycline (2 μg ml^−1^ or 1 μg ml^−1^), kanamycin (25 μg ml^−1^ or 5 μg ml^−1^), or spectinomycin (100 μg ml^−1^ or 25 μg ml^−1^), as required. PYE swarm plates contained 0.3% bacto agar. Site-directed mutagenesis of *cenK* and *cenR* was carried out using the primers CenKH273Afw, CenKH273Arev, CenRD60Efw, and CenRD60Erev, using the QuikChange protocol (Stratagene, La Jolla, California, United States). pKOC3 was constructed by PCR amplification of the tet^R^ cassette from pMR20 using the primers tet-fw and tet-rev, digestion with EcoRI, and ligation into the EcoRI site of pBluescript. Strains, plasmids, and primers used in this study are listed in Tables 4 and Figures S1–Figures S3.

**Table 4:**
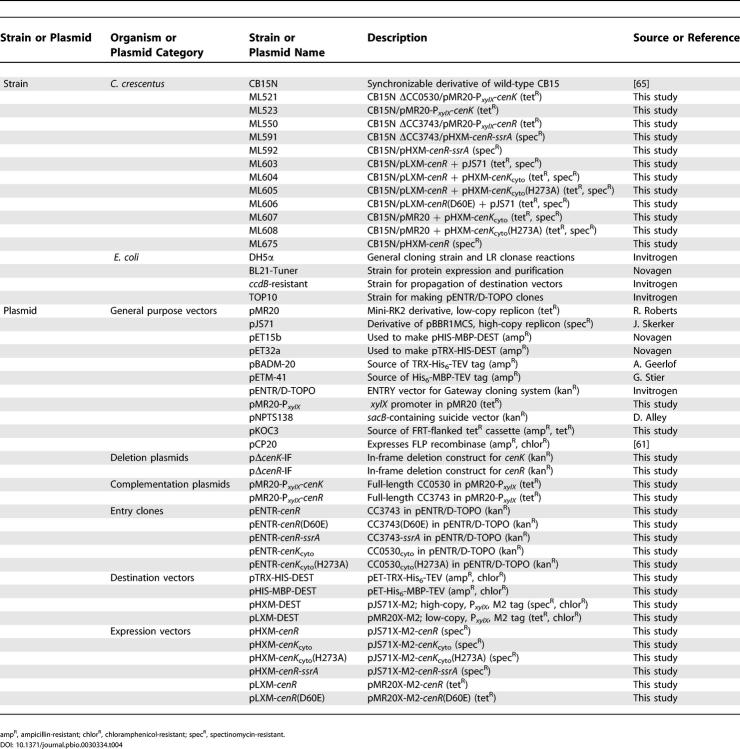
Strains and Plasmids

### Deletion of *C. crescentus* two-component genes

Response regulators and histidine kinases were identified by BLAST analysis of the *C. crescentus* genome sequence using known two-component protein sequences as input. For response regulators, sequences with BLAST *E*-values less than 0.01 were inspected for presence of the conserved residues D12, D13, D57, T87, and K109, where numbering is for *E. coli* CheY [10]. In sum, 44 response regulators were identified; these include two which may not be phosphorylated owing to mutation of one of the five highly conserved residues: CC3100 and CC0612. For histidine kinases, sequences with BLAST *E*-values less than 0.01 were inspected for presence of the conserved H-, N-, D/F-, and G-boxes [10]. Two histidine kinases, CC0433 and CC0594, are CheA-like and have a P1 domain instead of the usual H-box. Nine histidine kinases are members of the newly identified HWE group [58] and lack the F-box (CC0629, CC0836, CC1683, CC2554, CC2909, CC3048, CC3058, CC3170, and CC3560).

Deletion strains were generated by a long-flanking homology procedure and two-step recombination (see Figure S1) [59,60]. Complete lists of primers used are in Table S2. Regions of homology flanking each gene to be deleted were amplified in 50-μl reactions by PCR using the following conditions: 40 ng CB15N genomic DNA, 50 μM each dNTP, 100 nM each primer (P1 + P2a or P3a + P4), 1X Pfu Turbo buffer, 1.25 U Pfu Turbo polymerase (Stratagene), 2% DMSO, and 60 mM Betaine. For each reaction, 35 cycles of the following sequence were run: 94 °C for 1 min, 55 °C for 1 min, and 72 °C for 5 min. Reactions included a pre-incubation at 94 °C for 5 min, and concluded with a 10-min extension at 72 °C. Products were then re-amplified using identical conditions, but with primers P1 and P2b or P3b and P4. This produced final regions of flanking homology that were gel-purified (Qiagen, Valencia, California, United States) and used to amplify a tet^R^ cassette by PCR: 50 μM each dNTP, 100 nM P1 primer, 100 nM P4 primer, the products of the flanking homology PCRs, 1 mM MgCl_2_, 1X Taq buffer, 2% DMSO, 60 mM Betaine, 2.5 U Taq (Invitrogen, Carlsbad, California, United States), 0.5 U Pfx (Invitrogen), and 200 ng of the KpnI-SacI fragment of pKOC3 containing the tet^R^ cassette. Cycling comprised pre-incubation at 94 °C for 2 min; followed by ten cycles of the sequence 94 °C for 30 s, 53 °C for 30 s, and 72 °C for 8 min; followed by 20 cycles of the sequence 94 °C for 30 s, 53 °C for 30 s, and 72 °C for 8 min plus 20 additional seconds per cycle; followed by 72 °C for 10 min. Final PCR amplicons were gel-purified, blunted using the End-IT kit (Epicentre, Madison, Wisconsin, United States) and ligated into pNPTS138. Ligations were transformed into DH5α and positive colonies selected by blue/white screening. Plasmids from white colonies were verified by restriction digest with BamHI and HindIII or by sequencing. We term these plasmids “knockout plasmids” and name each according to the nomenclature pKO-CCXXXX, where CCXXXX is the unique GenBank identifier for the gene to be deleted. Knockout plasmids were transformed into CB15N by electroporation, and first integrants selected by plating on PYE containing oxytetracycline. Colonies were innoculated into liquid PYE medium with oxytetracycline and grown for 12–16 h. Five microliters of each culture was then plated on PYE plates containing oxytetracycline and sucrose. Colonies were screened for tetracycline resistance and kanamycin sensitivity to identify deletion strains. Proper construction of the gene deletion was also verified by two PCRs: one used tet-conf and a primer specific to the chromosomal region of the deleted gene (Pconf) and the other used sacBfw and sacBrev to verify loss of the *sacB* gene.

The first six and last 12 codons of each gene deleted were left intact to protect against disruption of possible regulatory signals for adjacent genes. For genes at the beginning of an operon, the presence of a tet^R^ cassette may lead to polar effects, but the majority of mutants found to have phenotypes (see Figure 2; Tables 1 and 2) were encoded as single genes and hence would not exhibit polarity. For simplicity we refer to all mutant strains by the gene disrupted, whether in a predicted operon or not. For mutants of interest that may suffer from polar effects, the tet^R^ cassette can be removed, as done for CC0530 and CC3743 (see below). The cassette is flanked by two direct repeats (FRT sites) such that expression of the FLP recombinase catalyzes removal of the tet^R^ cassette, leaving behind an in-frame deletion construct [61]. Each deletion strain was also engineered to incorporate two unique 20mer bar codes, each of which is not found elsewhere in the *C. crescentus* genome. The bar code sequences were adapted from the *S. cerevisiae* deletion project [40,41], enabling similar high-throughput phenotypic characterization of deletion strains using high-density microarrays with probes complementary to the bar codes (M. T. L., unpublished data).

### Generation of pENTR clones for response regulators and histidine kinases

Strains for expression and purification of His_6_-tagged proteins were generated using the Gateway high-throughput recombinational cloning system (Invitrogen). For each response regulator, the entire gene was amplified by PCR, using reverse phase cartridge purified primers (Sigma-Genosys, St. Louis, Missouri, United States). PCR reactions contained 60 mM Betaine, 2% DMSO, 1X Pfu buffer, 50 μM each dNTP, 75 ng CB15N genomic DNA, 10 pmol each primer, and 1.25 U Pfu Turbo (Stratagene). Reactions were incubated at 95 °C for 5 min, followed by 35 cycles of 95 °C for 1 min, 55 °C or 58 °C for 1 min, and 72 °C for 5 min, and finished by a 10-min extension at 72 °C. PCR amplicons were cloned into the pENTR/D-TOPO vector according to the manufacturer's protocol, and transformed into TOP10 competent cells (Invitrogen). Kanamycin-resistant (kan^R^) colonies were screened by colony PCR using M13F and M13R to verify the correct insert size. Positive clones were sequence-verified using M13F and M13R. In total, 76 Gateway adapted response regulator pENTR clones were generated for this study (32 for *E. coli* and 44 for *C. crescentus*). Each clone generated is named pENTR-CCXXXX or pENTR-bXXXX, where CCXXXX and bXXXX are the unique GenBank identifiers for the *C. crescentus* and *E. coli* genes, respectively. Cloning of histidine kinases was done identically to that of the response regulators, but the forward primer was designed to eliminate any transmembrane domains predicted by the SMART database (http://smart.embl-heidelberg.de). For complete primer lists, see Table S3.

### Destination vectors and recombinational cloning

Expression vectors were constructed and adapted for recombinational cloning using the Gateway vector conversion system (Invitrogen). Two plasmids (pTRX-HIS-DEST and pHIS-MBP-DEST) were derived from the IPTG-inducible pET32a and pET15b vectors (Novagen. Madison, Wisconsin, United States). To construct pTRX-HIS-DEST, a MscI-XhoI fragment of pBADM-20 (EMBL protein purification and expression facility) was used to replace the same region of pET32a. This resulting clone was digested with NcoI and blunted with T4 DNA polymerase, and the RfA Gateway cassette was cloned into this site. To construct pHIS-MBP-DEST, an XbaI-BamHI fragment of pETM-41 (EMBL protein purification and expression facility) was first cloned into pET15b. The resulting clone was digested with NcoI and blunted with T4, and the RfA cassette was cloned into this filled-in site. The vectors were designed to generate an N-terminal fusion to thioredoxin-His_6_, or His_6_-maltose binding protein, followed by the TEV protease cleavage site. Two other destination vectors were constructed for inducible expression in *C. crescentus* based on the low-copy (pMR20) and high-copy (pJS71) plasmids. These plasmids utilize the promoter region of the *xylX* gene [62] and add an N-terminal M2 epitope tag (DYKDDDDK) to the gene of interest immediately after the start methionine. To construct pHXM-DEST, the *xylX* promoter region was amplified with the primers XYLM2fw and XYLM2rev and cloned into pJS71 as a SacI-SalI fragment. This clone was then digested with SalI and blunted with T4, and the RfB cassette (Invitrogen) was cloned into this site. pLXM-DEST was derived from pHXM-DEST by removing a SacI-KpnI fragment, and blunt cloning into the SacI site of pMR20. For both of these vectors, P*_xylX_* is in the opposite direction of the P*_lacZ_* promoter. Using Gateway LR clonase reactions to mediate site-specific recombination, pENTR response regulator and histidine kinase clones were recombined with these destination vectors to create expression clones for either protein purification or in vivo *C. crescentus* studies. Each 10-μl LR reaction contained: 50 ng of destination vector, ∼50 ng of pENTR plasmid DNA, 1X LR buffer, 3 U topoisomerase I, and 1 μl of LR clonase enzyme mix (Invitrogen). Reactions were incubated overnight at room temperature, transformed into chemically competent DH5α cells, and plated on LB with antibiotics as necessary. Colonies were tested for resistance to ampicillin (pTRX-HIS-DEST and pHIS-MBP-DEST), spectinomycin (pHXM-DEST), or tetracycline (pLXM-DEST), tested for sensitivity to kanamycin to ensure no carryover of pENTR DNA, and PCR verified with T7F and T7R for *E. coli* expression vectors or M13F and M13R primers for *C. crescentus* vectors. The resulting expression plasmids were called pTRX-HIS-CCXXXX, for the *C. crescentus* response regulators and pTRX-HIS-bXXXX, for the *E. coli* response regulators. Expression plasmids for the *E. coli* and *C. crescentus* histidine kinases have a parallel nomenclature.

### Protein expression and purification

Expression plasmid DNA was transformed into *E. coli* BL21-Tuner cells. Single colonies were grown in 500 ml of LB to OD_600_ ∼0.6 and fusion proteins induced by addition of 300 μM IPTG. Cells were grown at 37 °C prior to induction and then shifted to 30 °C for 4 h before harvesting by centrifugation at 10,800 *g* for 5 min. Cells were pelleted and stored at −80 °C until needed. Native purifications of His_6_-tagged proteins were performed using affinity chromatography with Ni-NTA agarose beads (Qiagen). All steps of the purification (except for elution) were performed in batch using 50-ml conical tubes. The following buffers were used for purification: lysis buffer (20 mM Tris-HCl [pH 7.9], 0.5 M NaCl, 10% glycerol, 20 mM imidazole, 0.1% Triton X-100, 1 mM PMSF, 1 mg/ml lysozyme, 125 units benzonase nuclease [Novagen]), wash buffer (20 mM HEPES-KOH [pH 8.0], 0.5 M NaCl, 10% glycerol, 20 mM imidazole, 0.1% Triton X-100, 1 mM PMSF), elution buffer (20 mM HEPES-KOH [pH 8.0], 0.5 M NaCl, 10% glycerol, 250 mM imidazole), and storage buffer (10 mM HEPES-KOH [pH 8.0], 50 mM KCl, 10% glycerol, 0.1 mM EDTA, 1 mM DTT). Each cell pellet was resuspended in 10 ml of lysis buffer, incubated at room temperature for 20 min, sonicated, and then centrifuged for 60 min at 30,000 *g* to generate a cleared lysate. His_6_-tagged proteins were bound to 1 ml of Ni-NTA agarose slurry, washed twice with 50 ml of wash buffer, and then loaded onto an Econo-column (Bio-Rad, Hercules, California, United States) for elution. Purified protein was eluted using 2.5 ml of elution buffer and loaded directly onto a PD-10 column (Amersham Biosciences, Piscataway, New Jersey, United States) that had been pre-equilibrated with storage buffer. If necessary, samples were filtered with a 0.2-μm HT Tuffryn filter (Pall Gelman Sciences, East Hills, New York, United States), and then concentrated to approximately 1–10 mg/ml using Centricon YM-10 or YM-30 columns (Millipore, Billerica, Massachusetts, United States). All samples were filtered through an Ultrafree-MC (0.22 μm) spin filter (Millipore) and then aliquoted for storage at −80 °C. Protein concentrations were measured using Coomassie Plus Protein Assay Reagent and a BSA standard (Pierce Biotechnology, Rockford, Illinois, United States). An equal amount (500 ng) of each protein sample was analyzed by 12% SDS-PAGE to verify molecular weight and purity. Prior to phosphotransfer profiling, all response regulator concentrations were normalized against a 500-ng BSA standard using a ChemiImager 5500 and densitometry (Alpha Innotech, San Leandro, California, United States) (see Figure S5).

### Phosphotransfer profiling

Each purified kinase was autophosphorylated in storage buffer supplemented with 2 mM DTT, 5 mM MgCl_2_, 500 μM ATP, and 5 μCi [γ^32^P]ATP (∼6,000 Ci/mmol, Amersham Biosciences). Reactions were allowed to proceed until equilibrium at 30 °C (15 min to 2 h depending on the kinase). Purified response regulators were diluted to a final concentration of 5 μM in storage buffer plus 5 mM MgCl_2_. Phosphotransfer reactions contained 5 μl of phosphorylated kinase and 5 μl of response regulator (2.5 μM final concentration of each) and were incubated at 30 °C. Reactions were stopped with 3.5 μl of 4X sample buffer (500 mM Tris [pH 6.8], 8% SDS, 40% glycerol, 400 mM β-mercaptoethanol) and stored on ice until loaded. The entire sample was loaded, without heating, on 10% Tris-HCl ready gels (Bio-Rad) and electrophoresed at room temperature for 50 min at 150 V. The dye front and unincorporated ATP was removed with a razor blade and the wet gel (still on the back glass plate) placed in a Ziploc bag and exposed to a phoshor screen for 1−3 h at room temperature. The screen was scanned with a Storm 860 imaging system (Amersham Biosciences) at 50 μm resolution. *E. coli* profiles consisted of three protein gels, which were scanned separately and the images stitched together for analysis. *C. crescentus* profiles consisted of four protein gels, and were analyzed in the same fashion.

### Estimation of kinetic preference

To estimate kinetic preference, we purified radiolabeled kinase by repeated washing with a Nanosep-30K column (Pall, East Hills, New York, United States). Autophosphorylation and phosphotransfer reactions were as described for phosphotransfer profiling, except that response regulators were diluted in storage buffer plus 5 mM MgCl_2_ plus 0.5 mg/ml bovine serum albumin. The final concentrations of kinase and regulator in the reaction were 2.5 μM and 0.25 μM, respectively. Kinetics of phosphotransfer were determined by quantifying bands using ImageQuant software (Amersham Biosciences). The fraction of phosphorylated response regulator was calculated by normalizing to the intensity of the band corresponding to kinase alone. These normalized values were plotted versus reaction time and used to estimate initial reaction velocities for cognate versus noncognate substrates.

### Depletion, overexpression, and coexpression strains

A xylose-inducible low-copy plasmid was generated by amplifying the *xylX* promoter region with XYLSACfw and XYLNCOrev and cloning into pMR20, to generate pMR20-P*_xylX_*. This plasmid contains a unique NcoI site engineered at the start codon of the *xylX* gene. We then amplified, by PCR, full-length versions of CC0530 *(cenK)* and CC3743 *(cenR)* flanked by NcoI and HindIII sites using the primers CenKNCOfw, CenKH3rev, CenRNCOfw, and CenRH3rev. The full-length *cenK* and *cenR* PCR products were cloned into pMR20-P*_xylX_*, to generate pMR20-P*_xylX_*-*cenK* and pMR20-P*_xylX_*-*cenR*.

Next, we produced in-frame derivatives of pKO-CC0530 and pKO-CC3743 (see above). Each of these plasmids was cotransformed into *E. coli* with pCP20, which contains an arabinose-inducible FLP recombinase gene [61]. Expression of the FLP recombinase, according to the methods of Datsenko and Wanner [61], led to recombination between the direct repeat FRT sites flanking the tet^R^ cassette. The resulting plasmids, pΔ*cenK*-IF and pΔ*cenR*-IF were sequenced to verify formation of an in-frame, markerless deletion construct. CB15N was then electroporated with pΔ*cenK*-IF and pΔ*cenR*-IF to generate kan^R^, sucrose-sensitive integrants. These first integrants were made electrocompetent and transformed with the complementing plasmids pMR20-P*_xylX_*-*cenK* or pMR20-P*_xylX_*-*cenR* described above, or a vector control pMR20. Single colonies from each transformation were then grown overnight in PYE containing oxytetracycline and 0.03% xylose (for *cenK*) or 0.0003% xylose (for *cenR*). After overnight growth, 1 μl was plated for counter-selection on PYE containing 3% sucrose and oxytetracycline. Using markerless deletion constructs, there were three possible outcomes for colonies from counter-selection: (i) regeneration of the wild-type allele, (ii) generation of an in-frame deletion, or (iii) *sacB* inactivation without plasmid excision. Ninety-six sucrose^R^ colonies were picked, tested for kanamycin sensitivity, and analyzed by PCR using CenKconf_fw plus CenKconf_rev or CenRconf_fw plus CenRconf_rev. Colonies that were kanamycin sensitive and yielded a single PCR band of the expected size indicated colonies with an in-frame deletion. These procedures produced the depletion strains ML521 and ML550. ML521 grew only in the presence of xylose. As strain ML550 formed single colonies on plates supplemented with either glucose or xylose, we constructed a destablized version of CenR by adding a C-terminal *ssrA* tag (AANDNFAEEFAVAA) using the primers CenRfw and CenRssrArev. This construct was cloned into pENTR/D-TOPO and recombined into pHXM-DEST by the LR clonase reaction, to generate pHXM-*cenR-ssrA*. This plasmid was used to construct strain ML591 (Δ*cenR* + pHXM-*cenR-ssrA*), as described above for ML550. ML591 grew only in the presence of xylose.

To study the depletion strains ML521 and ML591, overnight cultures grown in xylose (0.03% for ML521 and 0.3% for ML591) were washed twice with PYE and diluted back 1:20,000 in PYE plus 0.1% glucose for CenK or 1:1,000 for CenR. As a control, each strain was also diluted 1:20,000 in PYE plus xylose. After 12 h of depletion, cells had grown to a sufficient density to be measured, and OD_600_ was monitored for an additional 8 h before samples were fixed for light and electron microscopy.

For overexpression and coexpression studies, CenK_cyto_, CenR, CenR(D60E), and CenK_cyto_H273A expression vectors were generated by Gateway cloning (see Tables 4 and S1). These vectors were electroporated into CB15N and selected with oxytetracyline, spectinomycin, or both as necessary. Overnight cultures were washed in PYE and diluted 1:50 in PYE supplemented with glucose or xylose. Samples were monitored by OD_600_ and fixed for light microscopy. Overexpression of CenR on pHXM-*cenR* was performed with 0.3% xylose, whereas all other experiments were done using 0.03% xylose.

### Microscopy

*C. crescentus* cells were grown to mid-log phase, fixed by addition of 0.5% paraformaldehyde in PBS, washed, and concentrated with PYE medium. Samples were deposited on microscope slides coated with 0.1% poly-L-lysine. Differential interference contrast images were obtained with a Zeiss (Oberkochen, Germany) Axioskop2 equipped with a 100× Plan-NEOFLUAR (NA 1.3) objective and an AxioCam monochrome CCD camera controlled by Axiovision 4.1 software. For field emission scanning electron microscopy (FESEM), cells were pelleted at 10,000 *g* and resuspended in fix solution (0.1 M sodium cacodylate buffer [pH 7.4], 2% glutaraldehyde, 0.5% paraformaldehyde, 7.5% sucrose). After concentrating, cells were deposited on a poly-L-lysine-coated glass coverslip, then post-fixed with 1% osmium tetroxide in 0.1 M sodium cacodylate buffer and 7.5% sucrose. After washing, samples were dehydrated with an ethanol series (10 min each of 50%, 70%, 90%, 95%, and 100%), critical-point dried by the CO_2_ method (AutoSamdri 850, Tousimis, Rockville, Maryland, United States), and sputter coated with an approximately 5-nm layer of gold/palladium (Desk II, Denton Vacuum, Moorestown, New Jersey, United States). Cells were imaged with a LEO 982 FESEM (Zeiss) using a SE-INLENS detector operated at 2.0 keV.

## Supporting Information

Figure S1 Diagram of Two-Step Deletion Procedure Deletion constructs were generated with a splice-overlap extension protocol using six different primers (see Table S2). Each gene is disrupted by a tet^R^ cassette and is flanked by approximately 800 bp of upstream (LFH) and downstream (RFH) flanking homologous DNA for efficient recombination. A suicide vector for each gene to be deleted contains a kan^R^ gene and a sucrose counter-selectable *sacB* marker. A two-step recombination procedure results in the generation of a chromosomal deletion strain (tet^R^, kan^S^, sucrose^R^). This method also allows the identification of putative essential genes (tet^R^, kan^R^, sucrose^R^).
(6.5 MB TIF).

Figure S2 Estimation of Kinetic Preference (A and B) Time courses for phosphorylation of OmpR and CpxR by EnvZ. In our phosphotransfer profiling (Figure 4C), OmpR and CpxR were both phosphorylated at the 60-min time point, but only OmpR was phosphorylated at the 10-s time point.
(C) Plot of normalized PhosphorImager counts for OmpR and CpxR phosphorylation based on a quantification of the gels shown in (A) and (B). Initial velocities (v_0_) were determined by measuring the slope (counts/second) for OmpR between 0 and 5 s, and for CpxR between 0 and 4,000 s.
(D and E) Time courses for phosphorylation of CC1182 and CC2931 by CC1181. In our profiling, both CC1182 and CC2931 were phosphorylated by CC1181 at 60 min, but only CC1181 was phosphorylated at 10 s.
(F) Plot of normalized PhosphorImager counts for CC1182 and CC2931 phosphorylation based on a quantification of the gels shown in (D) and (E). Initial velocities were determined for CC1182 between 0 and 10 s, and for CC2931 between 0 and 4,000 s. See Materials and Methods for experimental details. To estimate kinetic preference, in the Michaelis–Menton formalism, at substrate concentrations much less than *K*_m_: v_0_ ≍ [E][S] (*k*_cat_/*K*_m_). In these time courses, we tested response regulators at a concentration of 0.25 μM, which is approximately 10-fold lower than the typical *K*_m_ of a kinase–regulator pair [63]. As the enzyme and substrate concentrations used in these time courses were identical, the ratio of *k*_cat_/*K*_m_ for a cognate substrate relative to a noncognate substrate is estimated simply by: v_0,congate_/v_0,non-cognate_. From a quantification of our time course data, this ratio is approximately 1,330 for OmpR relative to CpxR and approximately 1,760 for CC1182 relative to CC2931. An in-depth kinetic characterization of individual kinases would be necessary to precisely determine *k*_cat_ and *K*_m_, but the order of magnitude is 10^3^. Moreover, in each case, this 10^3^-fold preference is for a cognate response regulator relative to the next best substrate, suggesting that other regulators are separated by an even greater kinetic gap.
(4.0 MB TIF).

Figure S3 Multiple Sequence Alignment of CenR Orthologs Putative CenR orthologs were identified by reciprocal best BLAST analysis. CenR proteins are highly conserved in the alpha subdivision of proteobacteria (*C. crescentus* CB15, *Agrobacterium tumefaciens* C58, *Sinorhizobium meliloti* 1021, *Mesorhizobium loti* MAFF303099, *Brucella melitensis* 16M, *Rhodopseudomonas palustris* CGA009, *Bradyrhizobium japonicum* USDA 110, *Rhodobacter sphaeroides* 2.4.1, *Silicibacter pomeroyi* DSS-3).
(5.4 MB TIF).

Figure S4 Multiple Sequence Alignment of CenK Orthologs Putative CenK orthologs were identified by reciprocal best BLAST analysis. CenK proteins are highly conserved in the alpha-subdivision of proteobacteria (*C. crescentus* CB15, *Agrobacterium tumefaciens* C58, *Sinorhizobium meliloti* 1021, *Mesorhizobium loti* MAFF303099, *Brucella melitensis* 16M, *Rhodopseudomonas palustris* CGA009, *Bradyrhizobium japonicum* USDA 110, *Rhodobacter sphaeroides* 2.4.1, *Silicibacter pomeroyi* DSS-3).
(2.3 MB TIF).

Figure S5 Purified *C. crescentus* and *E. coli* Response Regulators (A) Thirty-two *E. coli* response regulators were purified as thioredoxin-His_6_ fusion proteins.
(B) Fourty-four *C. crescentus* response regulators were purified as thioredoxin-His_6_ fusion proteins. Approximately 500 ng of purified protein was analyzed by 12% SDS-PAGE. The predicted molecular weights can be found in Table S3. Only one response regulator, *E. coli* FimZ, was not purified in a soluble form (no band of the correct weight is found in this lane). A molecular weight ladder is labeled in kilodaltons.
(3.2 MB TIF).

Table S1 Primer Names and Sequences Used for Plasmids Constructed in This Study (15 KB XLS).

Table S2 Primers for Deletion of *C. crescentus* Two-Component Signal Transduction Genes For each gene to be deleted, six primers were required (P1, P2a, P2b, P3a, P3b, and P4) plus one gene-specific confirmation primer (Pconf) (see Figure S1). The resulting deletion constructs are called “pKO-CCXXXX” where CCXXXX is the unique GenBank identifier number.
(66 KB XLS).

Table S3 Primers for pENTR Clones of Histidine Kinases and Response Regulators List of primers used to clone 44 *C. crescentus* response regulators, and 32 *E. coli* response regulators. Each resulting pENTR clone is called pENTR-CCXXXX or pENTR-bXXXX for *C. crescentus* and *E. coli* genes, respectively. Three *E. coli* histidine kinases and four *C. crescentus* histidine kinases were also cloned, and the primers used are listed.
(33 KB XLS).

### Accession Numbers

The GenBank (http://www.ncbi.nlm.nih.gov/Genbank/) accession numbers for the CenR orthologs discussed in this paper are *Agrobacterium tumefaciens* C58 (Atu2763), *Bradyrhizobium japonicum* USDA 110 (bll0620), *Brucella melitensis* 16M (BMEI0066), *C. crescentus* CB15 (CC3743), *Mesorhizobium loti* MAFF303099 (MBNC03001136), *Rhodobacter sphaeroides* 2.4.1 (Rsph03000729), *Rhodopseudomonas palustris* CGA009 (RPA0283), *Silicibacter pomeroyi* DSS-3 (STM1w01002705), and *Sinorhizobium meliloti* 1021 (SMc03820). GenBank accession numbers for the CenK orthologs are *Agrobacterium tumefaciens* C58 (Atu0388), *Bradyrhizobium japonicum* USDA 110 (bll8095), *Brucella melitensis* 16M (BMEI1648), *C. crescentus* CB15 (CC0530), *Mesorhizobium loti* MAFF303099 (MBNC03004238), *Rhodobacter sphaeroides* 2.4.1 (Rsph03002719), *Rhodopseudomonas palustris* CGA009 (RPA0635), *Silicibacter pomeroyi* DSS-3 (STM1w01001404), and *Sinorhizobium meliloti* 1021 (SMc01716).

## Abbreviations

kan^R^: kanamycin resistant/resistance
PYE: peptone yeast extract
sucrose^R^: sucrose-resistant/resistance
tet^R^: tetracycline resistant/resistance
